# Alternatives to project-specific consent for access to personal information for health research: Insights from a public dialogue

**DOI:** 10.1186/1472-6939-9-18

**Published:** 2008-11-19

**Authors:** Donald J Willison, Marilyn Swinton, Lisa Schwartz, Julia Abelson, Cathy Charles, David Northrup, Ji Cheng, Lehana Thabane

**Affiliations:** 1 Department of Clinical Epidemiology & Biostatistics, McMaster University, Hamilton, Canada; 2 Centre for Evaluation of Medicines, St. Joseph's Healthcare, Hamilton, Canada; 3 School of Nursing, McMaster University, Hamilton, Canada; 4 Department of Philosophy, McMaster University, Hamilton, Canada; 5 Centre for Health Economics and Policy Analysis, McMaster University, Hamilton, Canada; 6 Institute for Social Research, York University, Toronto, Canada; 7 Biostatistics Unit, St. Joseph's Healthcare, Hamilton, Canada

## Abstract

**Background:**

The role of consent for research use of health information is contentious. Most discussion has focused on when project-specific consent may be waived but, recently, a broader range of consent options has been entertained, including broad opt-in for multiple studies with restrictions and notification with opt-out. We sought to elicit public values in this matter and to work toward an agreement about a common approach to consent for use of personal information for health research through deliberative public dialogues.

**Methods:**

We conducted seven day-long public dialogues, involving 98 participants across Canada. Immediately before and after each dialogue, participants completed a fixed-response questionnaire rating individuals' support for 3 approaches to consent in the abstract and their consent choices for 5 health research scenarios using personal information. They also rated how confident different safeguards made them feel that their information was being used responsibly.

**Results:**

Broad opt-in consent for use of personal information garnered the greatest support in the abstract. When presented with specific research scenarios, no one approach to consent predominated. When profit was introduced into the scenarios, consent choices shifted toward greater control over use. Despite lively and constructive dialogues, and considerable shifting in opinion at the individual level, at the end of the day, there was no substantive aggregate movement in opinion. Personal controls were among the most commonly cited approaches to improving people's confidence in the responsible use of their information for research.

**Conclusion:**

Because no one approach to consent satisfied even a simple majority of dialogue participants and the importance placed on personal controls, a mechanism should be developed for documenting consent choice for different types of research, including ways for individuals to check who has accessed their medical record for purposes other than clinical care. This could be done, for example, through a web-based patient portal to their electronic health record. Researchers and policy makers should continue to engage the public to promote greater public understanding of the research process and to look for feasible alternatives to existing approaches to project-specific consent for observational research.

## Background

Internationally, the secondary use of existing personal health information for research purposes is intensifying. While administrative datasets continue to have an important role in a variety of health research, increasingly researchers are turning to clinical records, as they become available in electronic format. These clinical records provide a much richer source of data than is available through administrative records. In addition, registries are being developed in many academic healthcare facilities to serve as data sources for a variety of future research needs.

The role of consent in the secondary use of health information for a variety of types of observational research involving the health record has been particularly contentious. Until recently, the policy discussion has focused on the circumstances under which a particular research protocol would be exempted from obtaining individual consent. More recent discussions have acknowledged a broader range of consent options involving: [1]

- opting-in (project-specific or a broad authorization for research use);

- opting-out (usually with some notification process); or

- use without the option of opting out.

The views of the public in this matter have been sought in several different countries and have been summarized in a previous paper [2]. Briefly, public attitudes on the need for and type of consent for research use of their health information are context-specific. Factors that influence consent choice include: the identifiability of the data [3,4]; whether there is any commercial element to the research [2]; the type of information being accessed [5]; and the trust that the information will be kept confidential [6].

In 2005, we surveyed the Canadian public on a spectrum of alternatives to conventional project-specific consent for research use of personal information, including: no use at all, prior individual consent for each use, prior broad authorization for different types of uses, notification with an opportunity to opt-out, and use without consent or notification. Findings indicated that the public values both privacy and health research and would be concerned if either of these impinged upon the other. The majority of the public was open to alternatives to conventional project-specific consent; however, there was no clear preferred approach to consent for use of personal information for health research [2].

Recognizing this is a complex and challenging topic, in the spring of 2005, we conducted a series of structured public dialogues across Canada as an alternative method to elicit public values and to work toward an agreement about a common approach to consent for research use of personal information. Public dialogues differ from focus groups. Focus groups are commonly used to gain richer insight into the reasons behind particular attitudes toward a product, service, or a concept. By contrast, public dialogues seek to find agreement on fundamental values-based choices and substantial consensus for policy directions [7]. While differences are neither suppressed nor ignored, there is a deliberate choice to build on the common ground rather than focus on differences. This paper presents findings from these dialogues and compares them with the findings from the public opinion survey to draw further insights about the nature of public values surrounding this topic and the ability to seek consensus regarding future policy directions.

## Methods

### Recruitment for dialogues

We convened seven day-long public dialogues: two in Vancouver, British Columbia, and in Montreal, Quebec, and one each in Hamilton, Ontario, Toronto, Ontario, and Halifax, Nova Scotia. We chose these cities to obtain broad regional representation from across Canada. Five of the dialogues were held in English and two in French. Participants in two of the dialogues were recruited through an invitation at the end of a public opinion survey of 1230 Canadians that we had conducted on the topic of privacy and access to personal information for health research [2]. The remainder were recruited through random-digit dialling within the vicinity of the target cities, by the Institute for Social Research, York University. Dialogue participants were provided an honorarium of $50. During recruitment, these participants were administered a small subset of questions from the original survey to determine comparability of this sample to the sample that participated in the full survey, with regard to socio-demographics and opinion on general attitudes toward privacy and health research.

### Background workbook

One week before the dialogues, participants received a workbook containing background information to facilitate an informed conversation. The workbook presented three general approaches to the role of consent in the use of personal information for health research as a starting point for their discussion:

- Approach 1: consent for each research use. This approach maximized individual choice. It also most closely reflects the current default assumption for secondary use of personal information generally: consent obtained in advance for a very specific use of the information.

- Approach 2: not requiring consent for research use of their information (called "assumed consent" in the dialogues). This approach maximized efficiency of research and represents the way information from the medical record has been used historically for quality improvement/system management, and for medical education. Participants were told there would be a notification system that one's information was being used for research purposes, with an option to opt-out of research use, but the onus was on the individual to do so.

- Approach 3: broad authorization for a range of future research uses, determined by the individual (called "broad *consent*" during the dialogues). Under approach 3, the individual opted *into *research use of their information, but authorization extended beyond individual projects, and allowed for excluding certain types of uses, and for future changing of one's consent choices in the future or withdrawing permission entirely.

The workbook presented arguments for and against each approach, without reference to any particular disease or application. For all research uses, the operating assumption was that all directly identifying information would be removed prior to use. Participants were advised that this would make it difficult, though not impossible, to re-identify individuals.

### The dialogues

The dialogues were carried out by the Canadian Policy Research Networks, using an adaptation of the method of Yankelovich [8]. The dialogues ran approximately six hours on either a Saturday or Sunday. The general approach was to have introductory material in a plenary session, with breakouts into smaller groups to deliberate on specific issues, followed by a plenary where each group presented a summary of its deliberations. In three dialogues, the number of participants was small enough that they did not break out into smaller groups for deliberations. All plenary sessions were audio recorded digitally and note-takers were assigned for the small group deliberations. At least one study co-investigator attended each of the sessions as a listener and a resource person, in the event there was a need for clarification over technical issues.

The first breakout session examined each of the three consent approaches in the abstract – i.e. independent of any specific disease, type of information, or type of research. Participants voiced what was desirable and any concerns with each approach. In the second, participants discussed their consent preference in three scenarios: (i) linking their health information with non-health information such as education and income levels; (ii) linking their health information with biological material and (iii) using their health information for commercial purposes. This was followed by a plenary discussion of how participant's consent preferences would change with each of the scenarios. Finally, participants broke into triads to discuss several possible safeguards and the impact these would have on their confidence that their information was being used responsibly. They then re-convened to discuss these in the larger group.

### Eliciting of consent choices

Prior to and immediately following the dialogues, participants completed a questionnaire (see Additional files 1 and 2) where they rated their level of support for each of the three general approaches to use of personal information for research, using a 7-point rating scale that varied from "dislike very much" to "like very much". They also indicated their consent choice for five different scenarios involving personal information for observational health research:

1. Research that tracks how doctors prescribe medications to give them feedback to help them improve the care they provide;

2. Research that tracks how doctors prescribe medications so drug companies can better target their advertising to doctors;

3. Research that looks at the relationship between health and work, education or income. To do this, information about work, education or income must be combined with information from the health record; and

4. Research that studies leftover tissue following surgery to better understand the cause of the disease – linking age, sex, diagnosis, and other medical conditions with the sample:

4.1. If the researchers have no plans to develop a commercial product, like a lab test, that is sold for profit

4.2. If the goal of the research is to identify a new test that could better diagnose if you had a condition that needed the surgery. The lab test would be sold for profit.

For each of the five scenarios above, consent options expanded on the three general approaches to include:

- My information should not be used for this purpose.

- My permission is needed each time before my information is used. (Approach 1)

- My general permission is needed. This could be for several different research studies. (Approach 3 – broad consent)

- My permission is not needed, but I want to know this is being done. (Approach 2 – assumed consent)

- There is no need for me to know. Just use it.

The first and last options gave the participants the opportunity to express an opinion that was either more restrictive or more permissive than the alternatives that we presented in the abstract discussion of consent approaches. Thus, response options were comprehensive and unconstrained by the three general approaches presented.

In the questionnaire administered immediately post-dialogue, participants were asked what would be the effect of a range of procedural and technical safeguards on their confidence that their information was being used responsibly. Then, they were asked to identify their consent choices for the same five scenarios presented in the pre-dialogue survey, assuming their top three safeguards were in place, and any other conditions they felt were necessary.

### Analyses

We tabulated participants' attitudes toward the three general consent approaches to using personal information for research. The 7-point scale was transformed, so that "strongly dislike" equalled "-3", a neutral opinion equalled "zero", and "strongly like" equalled "3". We tested the significance of the change in scores between pre- and post-dialogue using a parametric test (paired t-test) and used a non-parametric test (signed rank) to assess the robustness of the results. The criterion for statistical significance was set at alpha = 0.05 adjusted for multiple analyses using the Bonferroni method. All analyses were performed using SAS 9.1 (Cary, NC). As findings were equivalent, we report only the parametric statistics. We also examined what percent of participants changed their responses in the post-dialogue survey. We tested for any change between the post-dialogue and the pre-dialogue surveys and for a directional change in attitude from positive to negative or from negative to positive. Similarly, we tabulated consent choices across the 5 research scenarios and tested for changes in consent choices using paired t-test with signed rank test used to test the robustness of the results. We tested for differences pre-and post-dialogue between dialogue groups using Analysis of Variance (ANOVA) and Multivariate Analysis of Variance (MANOVA). We also inspected the patterns of responses using graphical techniques. Finally, we tabulated and ranked the proposed safeguards.

Transcripts from the dialogue had been coded for the purposes of writing up a report on the dialogues [9]. For this paper, we sought illustrative quotes that could inform the key findings from our quantitative analyses. To this end, codes related to the key themes derived from the quantitative analysis were reviewed by two members of the team and quotes were selected to provide more contextual information and insight into the meaning of the key themes regarding consent choices.

## Results

In total, 98 people took part in the dialogues. Participants were similar in age to the general population, but had a higher level of education and were more heavily represented by women (59% vs. 51%) than the general population. (Table 1) On ten key survey questions, dialogue participants who were drawn from the full survey (n = 21/98) expressed views that were somewhat more research friendly and less privacy concerned than the survey sample from which they were drawn. Attitudes toward privacy and health research were equivalent among source populations – i.e. those who completed the full telephone survey and those who answered the short telephone survey. (see Additional file 3)

**Table 1:** Participant demographics and comparison with general population

Category	Number of Participants (n = 98)	Participants (%)*	General Population^+ ^(%)*
Sex			
Female	58	59	51
Age			
20–39	37	38	37
40–59	35	36	39
≥ 60	26	27	23
Highest Level of Education			
High school or less	26	26	44
Some post-secondary	18	18.7	10
Completed post-secondary	42	42.7	41
Post graduate or professional degree	12	12.5	5
Other Categories			
Visible Minority	14	14	13
Aboriginal	2	2	4
Disabled	11	11	5

* Percentages may not add up to 100, due to rounding

^+ ^Total population demographic data: 2001 Census, Statistics Canada.

### Attitudes toward the three consent approaches in the abstract

**Figure 1:**
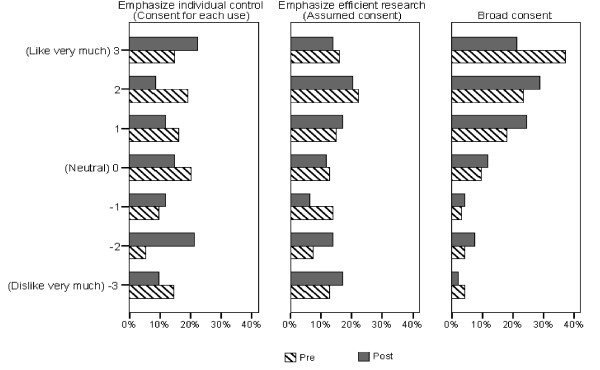
Comparison of Pre-Post Dialogue Ratings of Different Approaches to Consent.

Figure 1 compares participants' attitudes toward each of the three general approaches to consent for research use of one's personal information. Across the three approaches, broad consent (Approach 3) was regarded the most favourably, both pre- and post-dialogue. For Approaches 1 (consent for each use) and 2 (assumed consent), attitudes were almost evenly distributed across the spectrum – from extremely positive to extremely negative. For Approach 3 (broad consent), attitudes were skewed toward the positive.

Following the dialogue, participants generally rated all three approaches to consent somewhat lower. Only for broad consent did the change in rating approach statistical significance – and only when using the paired t-test prior to adjusting for multiple testing. (Table 2)

**Table 2:** Testing post-pre difference of approaches

Approach	Mean (SD)	Pre*	Post*	Post minus Pre	P-value for difference (paired t-test)**
1. Consent for each use	Mean (SD)	0.34 (1.95)	0.14 (2.07)	-0.21 (2.02)	0.315 (df = 95***)
2. Assumed consent	Mean (SD)	0.44 (1.99)	0.18 (2.10)	-0.29 (1.82)	0.122 (df = 96)
3. Broad Consent	Mean (SD)	1.55 (1.64)	1.21 (1.55)	-0.32 (1.54)	0.048 (df = 94)

* 7 Point scale where 3 = strongly like, 0 = neutral, & 3 = strongly dislike

** Bonferroni correction: Alpha = 0.017 (0.05/3)

*** df: Degree of freedom;

While there was shifting in response in approximately 55% of participants, an inflection in opinion (i.e. a change from a positive to a negative sentiment or vice-versa) occurred infrequently (8–23%), and was most stable for broad consent (92% no change). Our tests revealed no differences between dialogue groups in changes in ratings pre- vs. post-dialogue.

Participants generally liked certain aspects of each consent approach and disliked other aspects. From the discussions, the most frequently mentioned desirable feature of Approach 1 (consent for each use) was the opportunity for knowledge and communication that this approach facilitated – not just for purposes of control but also to better appreciate their contribution to the research.

"I like the idea that you would be given information about the specific research project because that gives you a sense of contributing to the community and what is needed for health care and it gives you a sense of, of being part of the picture without sacrificing your individual privacy." (Female, Toronto)

Participants also found Approach 1 to be particularly respectful of individuals.

" [It] was respectful I heard in two ways: One is because there's more awareness, but also it's the one that respects privacy the most was, was what several people said. So respectful of the information and in terms of knowing more how it would be used, but also respectful of the person's privacy." (Female, Vancouver)

At the same time, participants recognized that this approach is particularly burdensome for both researchers and for those whose data were to be used. They also recognized the increased potential for sampling biases using this approach.

Approach 2 (assumed consent) was recognized by participants as the most efficient approach and the least likely to produce biased results.

"There's a lot more incentive to do research, whereas the first approach would be very cost intensive and labour intensive." (Male, Toronto)

"I should think the research would be more, could be more accurate because they would have a wide group of people and most people aren't going to opt out." (Female, Toronto)

They also recognized the considerably reduced burden on the individuals whose consent would otherwise be sought. However, participants expressed concern over the onus placed on individuals if they wished *not *to participate.

"I think the, the less well or sicker individuals in the population just don't have the time or energy to consider becoming aware of things like opting out." (Female, Toronto)

They also expressed concern over the relative lack of individual knowledge or control under this approach, and the high potential for abuse.

"Is it really OK to just broadly take people's information without them being aware of it? And because of that, there is definitely room for abuse. I mean one would hope that that wouldn't happen but, you know, unfortunately there are abuse situations that do happen." (Female, Vancouver)

Approach 3 (broad consent) was seen by many to be a compromise between Approach 1 and Approach 2. It was seen to be less burdensome than Approach 1 but, as an *opt in *approach, it offered greater control than did Approach 2, and periodic renewal of their boundary setting.

"We liked this because we can change and set our own boundaries... and that can happen presumably at any stage in the, in the process." (Female, Vancouver)

The chief concern was how such a system of broad authorization might work.

The ability for individuals to maintain control was an important theme. Particularly in Approach 2 (assumed consent), participants emphasized the importance of having the opportunity to opt-out. However, regardless of the approach or the stated ability to opt-out at some future point, participants expressed concern over what might become of the data once released to the researcher.

**[Approach 1 – consent for each use] **"I don't think it's workable for the common good. You see, and the other issue raised with a central information gathering agency, you see it's open to anybody in the end. If you have enough money, if you throw enough money on it anybody can get your information once it is assembled in that type of fashion." (Male, Vancouver)

**[Approach 2 – assumed consent] **"Not being able to effectively opt out 'cause once you're in, how do you get the information back, it's already out the door? Like getting the cows back after they've left the barn." (Male, Vancouver)

**[Approach 3 – broad consent] **"... once you jot down, you check in this corner that it's OK to view your medical records or whatever it is, that broad consent, I mean I worked in a hospital with medical records, they, that can go all over the place and all the researchers do is they say 'Oh look, he checked that so we can do whatever we want.' and that's exactly what they do because they want to make it easier for themselves, understandably. We always want to get the, the red tape out of the way, and once that one consent is given then they can do this, this and this and this and nobody can stop them." (Male, Toronto)

### Consent choices for specific scenarios

**Figure 2:**
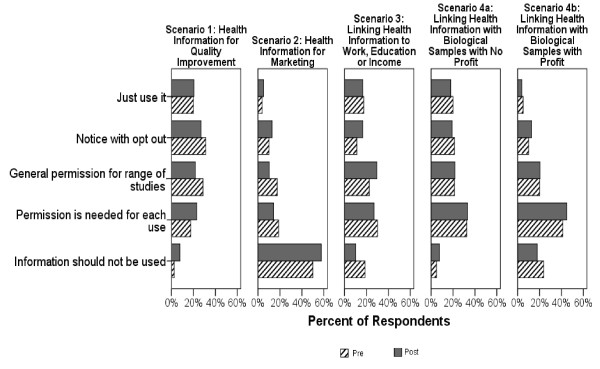
Figure 2 Pre and post-dialogue consent choices across research scenarios.

Figure 2 and Table 3 summarize participants' consent choices for each of the five scenarios. Across all scenarios, the response profile, in aggregate, was not substantively or statistically different pre- vs. post-dialogue. Consequently, we quote post-dialogue numbers in the following paragraphs. At the individual level, for all but one scenario, close to half of participants shifted their consent choice between the pre- and post-dialogue surveys. (Table 4) Shifting of response occurred approximately equally in both directions. The one exception was use of prescribing information for market research. Here, fewer individuals altered their response in the post-dialogue survey. Again, our tests revealed no differences between dialogue groups in changes in ratings pre- vs. post-dialogue.

**Table 3:** Testing post-pre dialogue change in consent choices across the five scenarios*

Scenario		Pre-Dialogue**	Post-Dialogue**	Difference	P-value for difference (paired t-test), df = 78 ***
A) Prescribing information for improving care	Mean (SD)	3.49 (1.08)	3.29 (1.25)	-0.21 (1.07)	0.096
B) Prescribing information for marketing research	Mean (SD)	1.99 (1.20)	1.94 (1.29)	-0.03 (0.88)	0.798
C) Linking work, education or income to individual's health record	Mean (SD)	2.79 (1.36)	3.03 (1.24)	0.19 (1.03)	0.104
D1) Linking individual's information with leftover tissue with no commercial use	Mean (SD)	3.18 (1.23)	3.06 (1.25)	-0.17 (1.27)	0.251
D2) Linking individual's information with leftover tissue with possible commercial use	Mean (SD)	2.31 (1.10)	2.40 (1.05)	0.06 (1.06)	0.595

* Due to an error in the Hamilton survey, the full consent scale was not offered. The option "Do not use" was not offered.

** Scores: 1 = do not use; 2 = consent for each use; 3 = broad authorization; 4 = notification with opt-out; 5 = use without notification;

*** Bonferroni correction: Alpha = 0.01 (0.05/5)

**Table 4:** Percentage of Participants who changed their responses to the five scenarios

Scenario	Change		
	More restrictive [x/n (%)]	No change [x/n (%)]	More permissive [x/n (%)]
A) Prescribing information for improving care	22/78 (28.21)	42/78 (53.85)	14/78 (17.95)
B) Prescribing information for marketing research	13/78 (16.78)	52/78 (66.67)	13/78 (16.78)
C) Linking work, education or income to individual's health record	16/78 (20.51)	39/78 (50.00)	23/78 (29.49)
D1) Linking individual's information with leftover tissue for non commercial product development	23/78 (29.49)	37/78 (47.44)	18/78 (23.08)
D2) Linking individual's information with leftover tissue for profitable product development	15/78 (19.23)	39/78 (50.00)	24/78 (30.77)

* Excluding Hamilton survey, for which the full consent scale was not offered.

**Use of prescribing information for quality improvement **yielded the most permissive response profile. Almost half of all participants opted for a passive process of either using the data without notification (21%) or notification with opt-out (27%). One-fifth opted for a broad consent and about 1/4 preferred their permission be sought for each use.

**Use of prescribing information for marketing research **yielded the most restrictive response profile. Almost 60% of respondents felt their information should not be used for this purpose at all. Another 14% felt their permission should be sought every time. Only 18% opted for a passive process of use without notification (5%) or notification with opt-out (13%).

**Linking work, education, or income with people's health information**. Opinions here covered the full spectrum of consent alternatives. About 10% felt this information should not be linked at all. About 1/4 preferred their permission be sought for each use. About 1/3 opted for broad consent and another 1/3 for a passive process of use without notification or notification and opt-out (17% each).

**Linking individuals' health information with leftover tissue **for non-commercial purposes showed a very similar response profile to the linkage with work, education, or income data. When linkage with leftover tissue involved development of a product for profit, consent choices became more restrictive, with the majority of people calling for either permission for each use (45%) or no such linkage at all (18%) with their information.

### Safeguards and Controls on Disclosure

**Figure 3:**
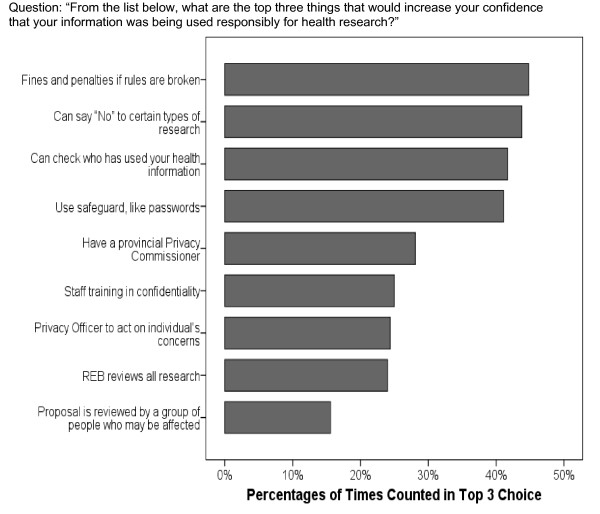
Top-ranked safeguards and controls.

Most of the 9 safeguards and controls identified in the post-dialogue survey either moderately or greatly increased people's confidence that their information would be used responsibly for research. When asked to identify the top three safeguards or controls, four stood out above the others: (1) fines and penalties for breaking rules; (2) the ability to say 'No' to use for certain types of research; (3) the ability to check who has used your health information; and (4) safeguards like passwords. (Figure 3)

Four of the five safeguards less often identified in the top three choices consisted of third-party controls over use of the information, including the existence of a provincial privacy commissioner, an institutional privacy officer, review of the proposed research by a research ethics board (REB), and review by a group of people who may be affected by the research. There was considerable discussion of REBs in particular, as they figured prominently in the workbook under each of the three general approaches to consent. Prior to the dialogues, most participants were unaware of the existence of REBs. While there were generally positive sentiments toward REBs, in several of the dialogues, concern was raised by some individuals that the REBs may over-ride their wishes to maintain control over use of their information.

There should be well-defined policies that guide the, the REB – especially if, if they're going to overturn what the individual consent, I mean the person who says "Well, we don't consent." and the REB says "Well, you know, go ahead anyways." There should be specific policies. (Male, Halifax)

But I think the way he put it was that there's a really big hole in, in the process. And that's the override that, in some situations, the researchers could simply – well, I shouldn't say "simply" 'cause they'd have to go through an REB – but they could decide to use information without, without actually having us as individuals give permission for it. (Female, Toronto)

These sentiments may have arisen in response to a statement in the workbook explaining that, under Approach 1 (consent for each project), an REB can permit the researcher to use personal health information without individual consent, so long as certain established conditions were met.

## Discussion and conclusion

### Dialogue participants were supportive but not passive

Overall, dialogue participants were very supportive of their de-identified health information being used for research. However, most do not wish to entirely let go of their ability to control use of their personal health information for research, even if direct identifiers are removed. This is consistent with our earlier survey of the Canadian population and survey and focus group work in other countries [2,6,10-13]. In addition, we learned from the dialogues that members of the public are sensitive to the practical implications of requiring consent – including the additional costs and the biases that can be introduced through an opt-in consent regime – and would not want to see this impede research. Some also see the consent process as more than an issue of control, viewing it also as an opportunity to see how their information was contributing to the public benefits that may ensue from research. This certainly reinforces the concept of consent as a transaction [14].

### No dominant consent choice emerged... with the exception of market research

We examined three general approaches to consent for research use of de-identified health information in the abstract and then considered specific scenarios with a full range of consent options. In the abstract, broad consent to a range of research was clearly preferred over project-specific consent or assumed consent. Based on this, one might have expected broad authorization to be a clear favourite when specific scenarios were presented. However, no more than 30% of dialogue participants chose this option under any of the five scenarios presented.

Indeed, no dominant consent choice emerged across the scenarios presented with one exception. Over half of dialogue participants (57%) felt their de-identified health information should not be used *at all *for marketing research (Scenario 2). This contrasts with laws in most jurisdictions internationally that permit commercial use of "anonymous" information and the common practice, globally, of compiling de-identified prescription data to ascertain physician prescribing patterns for marketing purposes [15].

Even in the presence of a clear public benefit (Figure 2, Scenarios 4a and 4b), the presence of some element of profit, resulted in a shift toward greater control over use of that information. When profit was introduced into the scenario, the percent of participants supporting use of the information without notification dropped substantially from 18% to 4% and the percent requiring project-specific consent grew from 32% to 44%. Further, those saying this information should not be used at all increased from 8% to 18%. While the response profile for this scenario was less restrictive than that observed for the pure marketing scenario, it still presents a challenge for research policy. It has been recognized that conventional project-specific consent for research using DNA databanks would render such research impracticable [16]. Much of this research is funded by the private sector and commercialization of discoveries made in the course of publicly funded research is strongly encouraged [17-19]. A more nuanced discussion over commercial interests in health research is warranted in future dialogues.

### Participants preferred personal controls

As for safeguards and controls, we note with interest that personal controls – consent and the ability to audit who has accessed one's information – were among the most commonly cited approaches that that improved people's confidence in the responsible use of their information for research. Third-party controls – e.g. research ethics boards, privacy officers, privacy commissioners, and panels of affected individuals – were nominated less often. In part, this may be due to lack of familiarity with these mechanisms. Yet, these mechanisms are key safeguards in our current system. In particular, research ethics boards have wide discretionary power to exempt specific research activities from requiring consent and the conditions under which this may occur. We note as well, regardless of consent regime, the high level of concern that was voiced over what happens to one's personal information once it *is *released to researchers.

### Limitations

We recruited our dialogue participants through random-digit dialling by an academically based polling firm. Twenty-one participants were recruited after completing a comprehensive attitude survey. The other 75 were asked a few general questions about health information privacy and about research use of their information. Our intention was to see if the dialogue process would be any different in groups that had an opportunity for prior consideration of this issue through the survey. Unfortunately, we were unable to recruit enough people from among those who participated in the full survey to allow this comparison. Indeed, participation rates in the dialogues were very low (approximately 2% of those completing the long survey and 4% of those completing the short survey). This raises the question of selection bias among dialogue participants. We noted earlier that participants had a higher level of education and were more heavily represented by women than the general population. Based on the common questions asked in both surveys, we were able to ascertain that dialogue participants were somewhat more research friendly and less privacy concerned than the population from which they were drawn. However, these differences were small.

Most public dialogues are associated with movement in position on the issue at hand by end of day [7,20]. In our dialogues, we saw little movement in opinion in aggregate. One may question whether this represents some failure of the dialogue process. We think not. Across all seven dialogue sessions, discussions were lively and constructive, displaying a spirit of mutual understanding. In addition, there was substantial movement in opinion at the individual level between the beginning and the end of the day-long dialogues (Tables 4 and 5). It seems the same evidence moved some individuals toward a more restrictive approach to use of their information for research and others toward a more permissive use. Based on this, we feel it is reasonable to conclude that we succeeded in being even-handed in the presentation of the issues at hand – or that our dialogue participants were sufficiently independent thinkers. In addition, our observation of little movement in aggregate is, in large part, consistent with findings from a recent public dialogue on the topic of biobanks in British Columbia [21].

**Table 5:** Percentage of Participants who changed their responses to the three general approaches to consent

Approach	Change	Any change [x/n (%)]	Inflection [x/n (%)]
1. Consent for each use	Less favourable	36/96 (37%)	11/96 (11%)
	No change	39/96 (41%)	77/96 (80%)
	More favourable	21/96 (22%)	8/96 (8%)
2. Assumed consent	Less favourable	29/97 (30%)	9/97 (9%)
	No change	42/97 (43%)	84/97 (87%)
	More favourable	26/97 (27%)	4/97 (4%)
3. Broad consent	Less favourable	32/95 (34%)	7/95 (7%)
	No change	42/95 (44%)	87/95 (92%)
	More favourable	21/95 (22%)	1/95 (1%)

### Policy implications

No one approach to consent satisfied even a simple majority of dialogue participants. Given this, as Canada moves toward developing a common inter-operable health record, consideration needs to be given to developing a system for a mechanism for documenting individuals' consent choices for research and other secondary uses of their personal health information, embracing a broader array of consent options than the current dichotomous alternatives of project-specific consent or exemption from consent. Given the high importance participants placed on being able to check who has accessed their medical record for purposes other than clinical care, this should also be considered. Both of these could be accommodated through a secure web-based portal that patients can use to access their health record, a technology that is gaining considerable attention [22-24].

We noted earlier that dialogue participants expressed concern over what happens to one's personal information once it *is *released to researchers – regardless of whether initial consent for the release of the information to the researcher was required. This highlights the importance of having trustworthy accountable systems for managing data. In part, this is a matter of ensuring that adequate access and security controls are in place in research facilities. Recent reports of lost or stolen health information involving negligence with regard to basic safeguards can readily undermine public confidence [25-28]. Regarding external accountability, in Canada, provincial information and privacy commissioners/ombudsmen currently have the authority to audit the practices of institutions that manage personal data. For practical reasons, this is usually a complaint-driven system. For larger data institutes, it is common practice to apprise these commissioners' offices of their data management practices in the absence of any complaint. Ideally, similar reporting systems should be in place for universities with smaller, more heterogeneous, data holdings. A practicable and meaningful reporting system remains an outstanding challenge for the research community.

Participants in our dialogues were largely unaware of the research process – particularly the ethic review process. For some, this raised concerns over the process. They also generally had difficulty articulating what it was about the commercial element to research that would cause them to desire greater control over use of their personal information when there was a profit element. Researchers and policy makers should continue to engage the public to promote greater public understanding of the research process – in particular, the public-private interface and the role of research ethics boards – and to better understand themselves how to best to respond to any concerns they may have. We should also continue to look for feasible alternatives to existing approaches to consent for observational research, when some form of consent is required.

## Competing interests

The authors declare that they have no competing interests.

## Authors' contributions

DW conceived and designed the study, participated in the dialogues, led the analyses and was the primary drafter of the manuscript. MS participated in the dialogues, conducted analyses, and reviewed and revised drafts of the manuscript. LS assisted in the design of the study, participated in the dialogues, reviewed analyses, and reviewed and revised drafts of the manuscript. JA, CC, and DN assisted in the design of the study, reviewed analyses, and reviewed and revised drafts of the manuscript. JC and LT participated in the analysis and interpretation of the data and reviewed and revised drafts of the manuscript. All authors read and approved the final manuscript.

## Pre-publication history

The pre-publication history for this paper can be accessed here:



## Supplementary Material

Additional File 1 **Pre-dialogue survey.** Questionnaire given to participants prior to the dialogues where they rated their level of support for each of the three general approaches to use of personal information for research and the 5 specific research scenarios. Click here for file

Additional File 2 **Post-dialogue survey.** Questionnaire given to participants following the dialogues where they rated their level of support for each of the three general approaches to use of personal information for research and the 5 specific research scenarios. Click here for file

Additional File 3 **Comparison of attitudes toward privacy and health research of dialogue participants recruited through the full and short surveys.** Comparison of attitudes toward privacy and health research of dialogue participants recruited through the full and short surveys. The purpose of this is to compare for selection biases. Click here for file
