# Supplementary material for: Alternatives to project-specific consent for access to personal information for health research: Insights from a public dialogue
Source: BMC Med Ethics. 2008 Nov 19;9:18. doi: 10.1186/1472-6939-9-18 (PMC2601042; doi:10.1186/1472-6939-9-18)
Supplement: Additional File 1 — Pre-dialogue survey. Questionnaire given to participants prior to the dialogues where they rated their level of support for each of the three general approaches to use of personal information for research and the 5 specific research scenarios. [file 1472-6939-9-18-S1.doc]

**PARTICIPANTS’ WORKSHEETS**

**Initial Thoughts**

**Citizens’ Dialogue on Privacy and the Use of Personal Information for Health Research in Canada**

The attached survey has a space at the top right corner so we can use a number code to identify your responses to our questions before and after today’s dialogue, so we can measure this change.

Please write your name on this cover sheet in the space below. Once we enter your unique code on the next page, we will tear off this cover sheet so your name does not appear anywhere on the survey.

Thank you, again, for participating in this dialogue. We look forward to hearing your views today.

Name ________________________________________________

(Please print)

**Initial Thoughts**

**Below we describe 3 different approaches for balancing privacy protection and allowing personal information to be used for health research.**

**Each of the approaches takes a different point of view or preference regarding privacy and health research in Canada. Please indicate how much you like or dislike each approach on a scale of 1 to 7 (1= dislike very much, and 7 = like very much; please circle one number for each choice)**

**1. Emphasize individual control through consent for each project.**

Information kept in any health record about you is your personal information. You should control what happens to it.

Using this approach:

- For each research project, you would be told who wants to use your information and how they are going to use it.
- You would need to give written permission for every project before your information can be used for any research project.

However, a Research Ethics Board could decide that your information could be used without your permission if it decides that the public benefit of the research is greater than the need to ask for consent and adequate safeguards are in place to protect the information

**1 2 3 4 5 6 7**

**Dislike very much Like very much**

**2. Emphasize efficient research by not requiring consent**

Personal information can be a great source of health research data. It should be easy for researchers to collect and use. It should not cost a lot of money to collect. The more information that can be collected for a study, the better it is for the research. Also, with more information, more questions can be answered to improve health for more people.

Using this approach, your information would be automatically available for research, unless you ask for it to be removed. There would be some sort of notice that your information is being used in this way.

**1 2 3 4 5 6 7**

**Dislike very much Like very much**

**3. Broad consent**

As more and more information gets collected on each one of us, the requests from researchers to use your information will grow.

Using this approach:

- You would give your written consent for your personal information to be used for the types of health research you are comfortable with. This could be any and all research, or you can put limits around how and why your information is used, and even what information cannot be used.

This allows the research process to be more efficient, but still gives you some control over use of your information.

**1 2 3 4 5 6 7**

**Dislike very much Like very much**

*4. Below are statements that describe different ways your health information may be used for research. In each case, your name, address and any other information that could directly identify you would be removed. Please check the answer that best matches your feelings about the use of your information for that kind of research.*

**A. Health research that tracks how doctors prescribe medications to give them feedback to help them improve the care they provide.**

- My information should not be used for this purpose.
- My permission is needed each time before my information is used.
- My general permission is needed. This could be for several different research studies.
- My permission is not needed, but I want to know this is being done.
- There is no need for me to know. Just use it.

**B. Health research that tracks how doctors prescribe medications so drug companies can better target their advertising to doctors?**

- My information should not be used for this purpose.
- My permission is needed each time.
- My general permission is needed. This could be for several different research studies.
- My permission is not needed, but I want to know this is being done.
- There is no need for me to know. Just use it.

**C. Health research that looks at the relationship between health and education or income. To do this, information about your education or income must be combined with information from your health record.**

- My information should not be combined for this purpose.
- My permission is needed each time.
- My general permission is needed. This could be for several different research studies.
- My permission is not needed, but I want to know this is being done.
- There is no need for me to know. Just use it.

**D. Health research that studies leftover tissue following surgery to better understand the cause of the disease.** To do this, your age, sex, diagnosis, and other medical conditions would be linked with the sample.

**If the researchers have no plans to develop a commercial product that is sold for profit, which statement best matches your feelings?**

- My information should not be used for this purpose.
- My permission is needed for each project.
- My general permission is needed. This could be for several different research studies.
- My permission is not needed, but I want to know this is being done.
- There is no need for me to know. Just use it.

**If the goal of the research is to identify a new test that could better diagnose if you had a condition that needed the surgery. The lab test would be sold for profit.**

- My information should not be used for this purpose.
- My permission is needed for each project.
- My general permission is needed. This could be for several different research studies.
- My permission is not needed, but I want to know this is being done.
- There is no need for me to know. Just use it.
