# Supplementary material for: Alternatives to project-specific consent for access to personal information for health research: Insights from a public dialogue
Source: BMC Med Ethics. 2008 Nov 19;9:18. doi: 10.1186/1472-6939-9-18 (PMC2601042; doi:10.1186/1472-6939-9-18)
Supplement: Additional File 2 — Post-dialogue survey. Questionnaire given to participants following the dialogues where they rated their level of support for each of the three general approaches to use of personal information for research and the 5 specific research scenarios. [file 1472-6939-9-18-S2.doc]

**PARTICIPANTS’ WORKSHEETS**

**Closing Thoughts**

**Citizens’ Dialogue on Privacy and the Use of Personal Information for Health Research in Canada**

**Like the survey you completed this morning,** this survey has a space at the top right corner so we can use a number code to identify your answers to our questions both before and after today’s dialogue. This will allow us to measure this change.

Please write your name on this cover sheet in the space below. Once we enter your unique code on the next page, we will tear off this cover sheet so your name does not appear anywhere on the survey.

Thank you, again, for participating in this dialogue. We hope you have found this to be a good experience.

Name ________________________________________________

(Please print)

McMaster University

**Now what do you think?**

Now that you have had a chance to consider different viewpoints and possible approaches that could be taken to protect privacy and allow personal information to be used for health research, what is your view?

**Please indicate how much you like or dislike each one on a scale of 1 to 7
(1= dislike very much, 7 = like very much). Circle the number that most closely matches your view.** If your viewpoint is based on a condition (e.g. “ I rate this approach at this level only if it also includes___________ or does not include_______”), please write that condition on the line immediately below your rating. If you need more room, please continue on the back of this sheet, including the question number the comments refer to.

**1. Emphasize individual control through consent for each project.**

**1 2 3 4 5 6 7**

**Dislike very much Like very much**

**On condition that:**

**a. it includes: ______________________________________________________________**

_______________________________________________________________________

_______________________________________________________________________

**b. it does not include:________**______________________________________________

__________________________________________________________________________

_______________________________________________________________________

**2. Emphasize efficient research by not requiring consent**

**1 2 3 4 5 6 7**

**Dislike very much Like very much**

**On condition that:**

**a. it includes: ______________________________________________________________**

_________________________________________________________________________________

**b. it does not include:________**______________________________________________

__________________________________________________________________________

**3. Broad Consent.**

**1 2 3 4 5 6 7**

**Dislike very much Like very much**

**On condition that:**

**a. it includes: ______________________________________________________________**

_______________________________________________________________________

_______________________________________________________________________

**b. it does not include:________**______________________________________________

__________________________________________________________________________

**4. Other issues and comments you want to share with us…**

**____________________________________________________________________________________________________________________________________________________________________________________________________________________________________________________________________________________________**

**5. How would each of the following controls affect your confidence that your information was being used responsibly for health research? Please circle the number on the 7-point scale that most closely matches your feeling, where 1 = “greatly reduces your confidence” and 7 = “greatly increases your confidence”**

|  | **Reduce No Increase**  **Greatly Effect Greatly** |
| --- | --- |
| a) Safeguards, like passwords on computer files, to prevent your information from being seen or used by someone who does not have permission. | **1 2 3 4 5 6 7** |
| b) Training for research staff in confidentiality. | **1 2 3 4 5 6 7** |
| c) Fines and penalties if any laws or rules are broken. | **1 2 3 4 5 6 7** |
| d) You can check who has used your health information for research, and the titles of the research studies. | **1 2 3 4 5 6 7** |
| e) You can say “No” to use of your information for certain types of research. | **1 2 3 4 5 6 7** |
| f) Research ethics boards review all research before that research can begin. | **1 2 3 4 5 6 7** |
| g) Any research proposal using personal information is also reviewed by a group of people who may be affected by that research. This group would identify concerns and make recommendations for change to the research. | **1 2 3 4 5 6 7** |
| h) There is a Privacy Officer in the organization that you can turn to if you have concerns about the use of your information. | **1 2 3 4 5 6 7** |
| i) There is a provincial Privacy Commissioner you can turn to if you are not satisfied with your discussion with the privacy officer. | **1 2 3 4 5 6 7** |
| j) Other _____________________________________ | **1 2 3 4 5 6 7** |
| **From the list above, what are the top 3 things that would increase your confidence that your information was being used responsibly for health research? (Please write down the corresponding letter below.)**  **(1)__________ (2) ____________ (3) ____________** | |

**6. What kind of consent would you want?**

*If the three controls you chose above were in place, which answer best matches your feelings about the use of your information for that kind of research?*

**A. Health research that tracks how doctors prescribe medications to give them feedback to help them improve the care they provide.**

- My information should not be used for this purpose.
- My permission is needed each time before my information is used.
- My general permission is needed. This could be for several different research studies.
- My permission is not needed, but I want to know this is being done.
- There is no need for me to know. Just use it.

**B. Health research that tracks how doctors prescribe medications so drug companies can better target their advertising to doctors?**

- My information should not be used for this purpose.
- My permission is needed each time.
- My general permission is needed. This could be for several different research studies.
- My permission is not needed, but I want to know this is being done.
- There is no need for me to know. Just use it.

**C. Health research that looks at the relationship between health and work, education or income. To do this, information about your work, education or income must be combined with information from your health record.**

- My information should not be combined for this purpose.
- My permission is needed each time.
- My general permission is needed. This could be for several different research studies.
- My permission is not needed, but I want to know this is being done.
- There is no need for me to know. Just use it.

**D. Health research that studies leftover tissue following surgery to better understand the cause of the disease.** To do this, your age, sex, diagnosis, and other medical conditions would be linked with the sample.

**If the researchers have no plans to develop a commercial product, like a lab test, that is sold for profit, which statement best matches your feelings?**

- My information should not be used for this purpose.
- My permission is needed for each project.
- My general permission is needed. This could be for several different research studies.
- My permission is not needed, but I want to know this is being done.
- There is no need for me to know. Just use it.

**If the goal of the research is to identify a new test that could better diagnose if you had a condition that needed the surgery. The lab test would be sold for profit.**

- My information should not be used for this purpose.
- My permission is needed for each project.
- My general permission is needed. This could be for several different research studies.
- My permission is not needed, but I want to know this is being done.
- There is no need for me to know. Just use it.

**Evaluation of the Dialogue**

**Finally, please take a few moments to indicate the extent to which you agree or disagree with each of the following statements. Please circle the number on the 7-point scale that most closely matches your feeling, where 1 = “totally disagree with the statement” and 7 = “totally agree with the statement” Our purpose in asking these questions is so that we can improve the process for future dialogue participants.**

|  | **Totally No Totally**  **Disagree Opinion Agree** |
| --- | --- |
| 1. The information package sent in advance provided helpful and interesting information. | **1 2 3 4 5 6 7** |
| 1. The phone calls during recruitment and after agreeing to participate gave helpful information. | **1 2 3 4 5 6 7** |
| 1. The registration process in the morning was efficient and friendly. | **1 2 3 4 5 6 7** |
| 1. The facilitators provided clear explanations, guidance and support throughout the day. | **1 2 3 4 5 6 7** |
| 1. The participant workbook was clear and contained relevant and useful information. | **1 2 3 4 5 6 7** |
| 1. Lunch and refreshments were satisfactory. | **1 2 3 4 5 6 7** |
| 1. The small group discussions were useful to me. | **1 2 3 4 5 6 7** |
| 1. There was sufficient opportunity for me to contribute and participate. | **1 2 3 4 5 6 7** |
| 1. Based on this experience, I would come to another citizens’ dialogue. | **1 2 3 4 5 6 7** |
| **10.** Overall, the one-day dialogue was worthwhile to me. | **1 2 3 4 5 6 7** |
| **11.** Additional comments about the day? Please write them here. Continue on the back of this sheet if needed. ___________________________________________________________ __________________________________________________________________________ | |
