# Supplementary material for: Alternatives to project-specific consent for access to personal information for health research: Insights from a public dialogue
Source: BMC Med Ethics. 2008 Nov 19;9:18. doi: 10.1186/1472-6939-9-18 (PMC2601042; doi:10.1186/1472-6939-9-18)
Supplement: Additional File 3 — Comparison of attitudes toward privacy and health research of dialogue participants recruited through the full and short surveys. Comparison of attitudes toward privacy and health research of dialogue participants recruited through the full and short surveys. The purpose of this is to compare for selection biases. [file 1472-6939-9-18-S3.doc]

| **Question** | **Source Population** | | **Dialogue Participants** | |
| --- | --- | --- | --- | --- |
|  | **Full Survey** | **Short survey** | **Full Survey** | **Short Survey** |
| **Number of respondents** | 1230 | 1828 | 21 | 75[[1]](#footnote-2)* |
| (All numbers below expressed as percents, rounded to the nearest digit.) | | | | |
| **How important is protecting the privacy of your personal information?** | | | | |
| not at all important | 2 | 2 | 0 | 7 |
| somewhat important | 23 | 13 | 33 | 12 |
| very important | 74 | 85 | 62 | 81 |
| don’t know | 1 | <1 | 5 | 0 |
| **In the past 5 years have you become more concerned or less concerned about protecting the privacy of your personal information? Or has there been no change?** | | | | |
| more concerned | 56 | 64 | 48 | 69 |
| less concerned | 2 | 1 | 5 | 3 |
| no change | 40 | 33 | 48 | 27 |
| don’t know | 2 | 1 | 0 | 1 |
| **In the electronic age, technology can be designed to ensure that privacy is respected.** | | | | |
| strongly agree | 35 | 37 | 33 | 39 |
| somewhat agree | 32 | 31 | 29 | 31 |
| somewhat disagree | 15 | 15 | 5 | 19 |
| strongly disagree | 13 | 11 | 29 | 9 |
| don’t know | 5 | 5 | 5 | 3 |
| **We need to get used to living with less privacy.** | | | | |
| strongly agree | 8 | 10 | 14 | 9 |
| somewhat agree | 8 | 17 | 14 | 20 |
| somewhat disagree | 19 | 22 | 24 | 19 |
| strongly disagree | 48 | 49 | 48 | 51 |
| don’t know | 3 | 2 | 0 | 1 |
| **More effort needs to be made to protect our privacy**. | | | | |
| strongly agree | 59 | 70 | 48 | 69 |
| somewhat agree | 32 | 22 | 29 | 17 |
| somewhat disagree | 6 | 5 | 5 | 12 |
| strongly disagree | 2 | 1 | 14 | 0 |
| Don’t Know | 2 | 2 | 5 | 1 |
| **Research that could be beneficial to people’s health is more important than protecting personal privacy** | | | | |
| strongly agree | 31 | 26 | 33 | 20 |
| somewhat agree | 37 | 37 | 43 | 36 |
| somewhat disagree | 17 | 17 | 5 | 25 |
| strongly disagree | 10 | 11 | 5 | 16 |
| don’t know | 5 | 8 | 14 | 3 |
| **People should allow their personal health information to be used for the benefit of society if this can be done without causing any harm to individuals.** | | | | |
| strongly agree | 40 | 38 | 52 | 39 |
| somewhat agree | 40 | 39 | 38 | 33 |
| somewhat disagree | 8 | 10 | 5 | 15 |
| strongly disagree | 9 | 10 | 5 | 11 |
| don’t know | 2 | 3 | 0 | 3 |
| **Everyone benefits if the privacy of individuals is respected.** | | | | |
| strongly agree | 66 | 72 | 76 | 80 |
| somewhat agree | 26 | 22 | 19 | 13 |
| somewhat disagree | 5 | 3 | 5 | 3 |
| strongly disagree | 1 | 2 | 0 | 3 |
| don’t know | 2 | 2 | 0 | 1 |
| **How concerned would you be if protecting your right to control access to your information made it difficult or impossible to conduct research?** | | | | |
| not at all | 8 | 9 | 10 | 11 |
| somewhat concerned | 57 | 57 | 62 | 55 |
| very concerned | 32 | 30 | 14 | 31 |
| respondent volunteers: “We can have both” | 1 | <1 | 5 | 0 |
| don’t know | 3 | 3 | 10 | 4 |
| **How concerned would you be if allowing health research made it very difficult to control how your health information was being used?** | | | | |
| not at all | 7 | 10 | 19 | 12 |
| somewhat concerned | 53 | 53 | 57 | 53 |
| very concerned | 38 | 34 | 19 | 33 |
| don’t know | 3 | 2 | 5 | 1 |

1. ^^ 2 participants do not have survey data. Therefore, total = 96 rather than 98 [↑](#footnote-ref-2)
